# Determinants of self-perceived burden among rheumatoid arthritis patients: a cross-sectional study

**DOI:** 10.1186/s42358-025-00473-4

**Published:** 2025-09-26

**Authors:** Yuping Tang, Yinghui Zhang, Lei Shi, Jieyu Zhang, Cailing Wang, Wei Wei, Liyu Zhang

**Affiliations:** 1 Department of Rheumatology and Immunology, The Second Hospital of Shanxi Medical University, Taiyuan, 030001 China; 2 Nursing Department, The Second Hospital of Shanxi Medical University, Taiyuan, 030001 China; 3 Intensive Care Unit, The Second Hospital of Shanxi Medical University, Taiyuan, 030001 China; 4 Department of General Surgery, The Second Hospital of Shanxi Medical University, Taiyuan, 030001 China

**Keywords:** Rheumatoid arthritis, Self-perceived burden, Activities of daily living, Socioeconomic factors, Patient-centered care, Cross-sectional study

## Abstract

**Purpose:**

To investigate the current status of self-perceived burden (SPB) among rheumatoid arthritis (RA) patients and analyze its influencing factors.

**Methods:**

A cross-sectional study was conducted with 140 RA patients recruited from four tertiary hospitals in Taiyuan city, Shanxi Province. The data was collected through structured questionnaires assessing: General Information Questionnaire, Self-Perceived Burden Scale (SPBS), Activities of Daily Living (ADL) Scale, Social Support Rating Scale(SSRS).Multiple linear regression were employed to identify key predictors of SPB.

**Results:**

From 112 distributed questionnaires, 104 were returned valid, with a 92.85% response rate. RA patients (40.38%) exhibited a moderate SPB, with a mean score of 29.10 ± 10.27. The primary concern was the emotional impact on caregivers, with the highest scores reflecting worry about caregiver burden (3.33±1.47). Multivariate linear regression analysis identified marital status (β = 0.19, *P* = 0.028), Activities of Daily Living (β=-0.26, *P* = 0.006), and family monthly income (β=-0.30, *P* = 0.002) as significant predictors of SPB in RA patients.

**Conclusions:**

The findings underscore the moderate but significant SPB in RA patients, with key influences being Activities of Daily Living, marital status, and economic factors. These insights are vital for tailoring supportive care and shaping public health strategies to better serve this patient population.

**Clinical trial registration number:**

Not applicable.

## Background

Rheumatoid arthritis (RA) is a chronic, inflammatory autoimmune disorder, characterized by joint inflammation, pain, stiffness, and eventual joint destruction [1, 2]. Globally, RA affects approximately 1% of the adult population, with women disproportionately affected at a rate approximately three- to five-fold higher than men [2]. A Chinese survey, noting the prevalence of RA as 1.02% in the country, which is among the 10 major chronic diseases, revealed an average annual direct cost of $1917.21 ± $2559.06 per RA patient [3]. The etiology of RA is complex and not fully understood, but it is believed to involve a combination of genetic predisposition, environmental triggers, and immune system dysfunction [4]. Current treatment measures for RA have evolved significantly, shifting from a palliative approach to one focused on disease modification. This paradigm shift includes the use of disease-modifying anti-rheumatic drugs (DMARDs), such as methotrexate and newer biological agents targeting specific inflammatory pathways like TNF-alpha inhibitors and IL-6 receptor antagonists [5, 6]. Therapeutic advances improved outcomes, reduced inflammation, slowed progression, and enhanced patients’ quality of life. However, long-term use may cause adverse effects, including gastrointestinal disturbances, bone marrow suppression, infections, and malignancies, requiring careful monitoring and personalized treatment.

RA imposes a considerable health burden globally. The personal burden stems from musculoskeletal defects, declining physical function, and reduced quality of life [7]. The socioeconomic burden encompasses not only direct medical expenses but also reduced work ability, dysfunction, and limited social engagement [7]. The psychological toll of RA cannot be overlooked. The chronic nature of the disease, coupled with physical limitations and potential social isolation, often leads to significant psychological distress, including depression, anxiety, and cognitive impairment [8, 9]. Thus, a holistic approach to RA management, integrating both physical and psychological interventions, is paramount to achieving optimal outcomes for patients.

Self-perceived burden (SPB) refers to the psychological experience of being a burden to others, impacting their lives due to the individual’s illness and the need for long-term caregiving [10]. SPB can lead to adverse emotional states such as depression, anxiety, guilt, and self-blame, affecting both the quality of life and treatment outcomes of patients [11, 12]. While existing research has primarily focused on general chronic diseases, including hypertension, coronary heart disease, diabetes, stroke, and malignancies, there is a lack of literature specifically addressing the self-perceived burden in RA. In one study, 386 elderly patients with RA in a hospital in Liaoning Province, China, were assessed using the Self-Perceived Burden Scale (SPBS) [13]. The findings indicated a favorable mediating effect of SPB in alleviating kinesiophobia among elderly individuals with RA. However, research in this area remains scarce and underreported.

Therefore, this study aims to investigate the current state of SPB among RA patients, identifying the key determinants that contribute to this burden, thereby laying a theoretical foundation for the development of targeted clinical nursing interventions.

## Methods

### Participants and study design

In this cross-sectional study, we employed a convenience sampling strategy to recruit patients diagnosed with RA who were admitted to 4 tertiary hospitals in Taiyuan, Shanxi Province, China, from April to October 2020. The inclusion criteria were: (1) fulfillment of the 2010 American College of Rheumatology/European League Against Rheumatism (ACR/EULAR) classification criteria for RA [14]; (2) possession of the capacity to read, comprehend, and effectively communicate; (3) voluntary consent to participate in the survey; and (4) attainment of the age threshold of 18 years or older. Exclusion criteria: (1) had concomitant other rheumatic autoimmune diseases; (2) exhibited severe systemic impairments affecting the cardiovascular, hepatic, renal, neurological, or endocrine systems; (3) suffered from psychiatric disorders; or (4) had undergone recent major surgical procedures or were experiencing unstable disease states. This research endeavor has been meticulously reviewed and granted approval by the Ethics Committee of the Second Hospital of Shanxi Medical University, under ethical approval number (2020) YX No. (100).

### Questionnaire introduction

#### General information questionnaire

The questionnaire encompasses demographic details and health metrics, including inquiries about gender, age, education, marital status, occupation, monthly income, disease duration, episode frequency, co-existing chronic diseases, and payment preferences.

#### Self-perceived burden scale (SPBS)

This validated tool is the sole instrument currently recognized for assessing the SPB among patients [15]. It consists of 10 items, scored on a 5-point Likert scale from “Always” (5 points) to “Never” (1 point). The total score (range from 10 to 50) reflects the level of perceived burden, with higher scores indicating a greater sense of burden. Notably, item 8 requires reverse scoring [16]. Scores below 20 indicate minimal burden, 20–29 suggest mild, 30–39 moderate, and 40 or more severe perceived burden. The scale’s reliability is confirmed with a Cronbach’s α of 0.85.

#### Activities of daily living (ADL) scale

Activities of Daily Living (ADL) encompass both Basic Activities of Daily Living (BADL) and Instrumental Activities of Daily Living (IADL). The Barthel Index is predominantly utilized for the assessment of Basic Activities of Daily Living (BADL).The Barthel Index (BI) evaluates daily living activities across 10 domains: transferring, walking, bowel/bladder control, dressing, eating, toileting, stair climbing, bathing, and grooming. BI scores range from 0 to 100, with higher scores signifying greater independence; 100 points indicate full independence, 61–99 suggest mild dependence, 41–60 moderate, and 40 or below severe dependence [1].

#### Social support rating scale (SSRS)

This scale measures social support levels across objective, subjective, and utilization dimensions. It includes 10 items scored from 12 to 66, with higher scores indicating stronger support. The scale is reliable and valid, with a test-retest reliability of 0.92 and item consistency coefficients from 0.89 to 0.94 [17].

### Data collection

The survey was carefully conducted by a team who explained its purpose to participants, obtained informed consent, and provided assistance for reading or visual challenges. Responses were recorded accurately, and completed questionnaires were checked for completeness and quality. Ultimately, a total of 112 questionnaires were distributed, yielding 104 valid and usable responses, translating into an impressive response rate of 92.85%.

### Sample size calculation

Sample size calculations, conducted with PASS 15.0 software considering an auto correlation of 0.87, determined a necessity for 89 participants to ensure statistical rigor at a significance level of α = 0.05 and power of 1-β = 0.9. To adjust for potential dropouts, the sample size was increased to 104, safeguarding the study’s integrity and statistical significance.

### Statistical analysis

Data analysis using SPSS 24.0 software meticulously presented continuous variables as means ± standard deviations and categorical variables as percentages. T-tests, ANOVA, and Pearson correlation analysis were employed to explore factors affecting SPB, followed by Multiple linear Analysis to pinpoint key determinants. A significance threshold of *P* < 0.05 was applied for hypothesis testing.

## Results

### Demographic characteristics

This study encompassed a total of 104 patients diagnosed with RA. The average age of the participants was 57.37 ± 11.78 years, and 78 (75%) were females. Furthermore, the duration of the disease varied substantially, spanning from 0 to 32 years, with a mean duration of 6.90 ± 8.09 years. The mean score for Social Support Rating Scale(SSRS) was 40.77 ± 6.02, and the mean score for Activities of Daily Living (ADL) was 86.25 ± 19.57.Detail demographic characteristics were showed in Table 1.

**Table 1:** General characteristics of study participants

Variables		Number	Percentage (%)
Gender	Male	26	25
	Female	78	75
Occupation	Worker	6	5.8
	Farmer	44	42.3
	Freelancer Office Worker	12	11.5
	Retired	8	7.7
		34	32.7
Education Level	Primary or Below	38	36.5
	Secondary/High School	48	46.2
	College or Above	18	17.3
Marital Status	Single	2	1.9
	Married	92	88.5
	Divorced	2	1.9
	Widowed	8	7.7
Religious Belief	Yes	12	11.5
	no	92	88.5
Medical Expense Payment	Urban Health Insurance New	46	44.2
	Rural Cooperative	58	55.8
Perceived Economic Sufficiency for Medical Expenses	Yes	50	48.1
	no	54	51.9
Disease Relapse Frequency	1 Time	22	21.1
	2 Times	22	21.2
	3 Times or More	44	42.3
	No Relapse	16	15.4

### SPB among RA patients

The SPBS among RA patients exhibited a broad spectrum, ranging from a minimal score of 13 to a peak score of 46, with an overall average of 29.10 ± 10.27, indicative of a moderate degree of self-reported burden. Notably, 40.38% of these patients reported experiencing a moderate level of SPB (Table 2). Among the various factors contributing to this burden, the item “Worrying that caregivers are assuming excessive responsibilities on my behalf” stood out with the highest median score of 3.33 ± 1.47, highlighting a prevalent concern. (Table 3). Age, disease duration, and level of social support showed positive correlations with self-perceived burden scores, whereas Activities of Daily Living (mean score 86.25 ± 19.57) demonstrated a significant negative correlation (Pearson’s *r*= -0.41, *p* < 0.002).RA patients of office workers (mean 38.00 ± 8.03), divorced (46.00±0.00), comorbidity with above 2 disease (37.71±7.88), income below 1000 (43.14±6.01) showed the highest SPB (all *P* < 0.05) (Table 4).

**Table 2:** Proportions of different levels of self-perceived burden among patients with rheumatoid arthritis

Self-perceived burden level	Number	Percentage
No Significant Burden	28	26.92%
Mild Burden	16	15.38%
Moderate Burden	42	40.38%
Severe Burden	18	17.32%
Total	104	100.00%

**Table 3:** Average scores for each item on the self-perceived burden scale among patients with rheumatoid arthritis

Item	Score
I worry that my caregiver’s health is affected by caring for me.	2.40±1.58
I worry that my caregiver’s health may not cope with the demands of caregiving.	2.00±1.32
I worry that my caregiver is taking on too much responsibility due to helping me.	3.33±1.47
I feel like I am a “big trouble” to my caregiver.	3.27±1.48
I feel that I am a burden to my caregiver.	3.23±1.49
I feel that my caregiver faces many challenges due to my illness.	3.21±1.44
I feel guilty about the demands I make on my caregiver.	3.06±1.50
I believe that my caregiver’s level of care exceeds their capacity.	2.54±1.55
I believe I have caused financial difficulties for my caregiver.	2.75±1.57
I worry that the costs of caring for me are too high.	3.29±1.53

**Table 4:** Univariate analysis of self-perceived burden in patients with rheumatoid arthritis

Variables	Number	SPBS score (Mean ± SD)	*R* value	t value	F value	*P* value
**Sex**				0.96		0.338
Male	26	30.77±11.00				
Female	78	28.54±9.96				
**Age**	104	29.10±10.22	0.01			0.923 ^a^
**Disease Duration**	104	29.10±10.27	0.03			0.822 ^b^
**Activities of Daily Living Score (Barthel Index)**	104	29.10±10.27	-0.41			0.002 ^c^
**Total Social Support Rating Scale Score**	104	29.10±10.27	0.03			0.743 ^d^
**Occupation**					3.75	0.007
Worker	6	18.33±4.03				
Farmer	44	29.55±10.97				
Freelancer	12	30.50±7.76				
Office Worker	8	38.00±8.03				
Retired	34	34.00±27.82				
**Religious Belief**				0.64		0.520
Yes	12	31.67±10.42				
No	92	28.76±10.32				
**Education Level**					0.17	0.680
Primary or Below	38	31.00±10.76				
Secondary/High School	48	29.17±9.79				
College or Above	18	24.89±10.39				
**Marital Status**					5.57	0.001
Single	2	14.00±0.00				
Married	92	28.39±9.75				
Divorced	2	46.00±0.00				
Widowed	8	36.75±8.92				
**Disease Relapse Frequency**					1.40	0.257
No Relapse	16	30.13±10.94				
1 Time	22	27.55±9.52				
2 Times	22	25.09±11.67				
3 Times or More	44	31.50±9.60				
**Comorbidity of Chronic Diseases**					3.07	0.036
None	60	28.73±10.92				
1 Type	26	26.00±9.76				
2 Types	14	37.71±7.88				
3 Types	2	±				
4 Types	2	±				
**Family Monthly Income**					2.96	0.024
Below 1000	14	43.14±6.01				
1000 ~ 2000	14	38.00±3.37				
2000 ~ 3000	24	40.08±5.21				
3000 ~ 4000	4	34.50±2.88				
Above 4000	46	41.78±6.70				
**Payment Method**				-1.69		0.097
Urban Health Insurance	46	26.43±11.37				
New Rural Cooperative	58	31.21±8.95				
**Relationship with Caregivers**					0.37	0.771
Spouse	80	28.38±9.80				
Son	12	33.17±10.99				
Daughter	8	29.75±11.47				
Parent	4	30.00±22.62				

**SPB**: Self-perceived burden. ^a^: P value for Pearson correlation analysis of age and SPB score. ^b^: P value for Pearson correlation analysis of disease duration and SPB score. ^c^: P value for Pearson correlation analysis of Activities of Daily Living and SPB score. ^d^: P value for Pearson correlation analysis of social support and SPB score

### Multivariate linear regression analysis on SPB

A multivariable linear regression analysis was performed to evaluate predictors of self-perceived burden scores in patients with rheumatoid arthritis. The independent variables included Activities of Daily Living score, occupation, marital status, number of comorbid chronic conditions, and monthly household income. The final regression model identified three significant predictors: marital status (β = 0.19, 95% CI: [0.25–4.41], p = 0.028), Activities of Daily Living score (β = -0.26, 95% CI: [-0.23–0.04], P = 0.006), and monthly household income (β = -0.30, 95% CI: [-3.41–0.79], p = 0.002). These factors collectively explained 31% of the variance in self-perceived burden scores (adjusted R² = 0.31).“(Table 5).

**Table 5:** Multiple linear regression analysis of self-perceived burden in rheumatoid arthritis patients

Variables	Unstandardized coefficient(B)	Standard error(SE)	Standardized coefficient	T value	*P* value	95%CI
Marital status	2.33	1.04	0.19	2.23	0.028	0.25–4.41
Activities of Daily Living	-0.13	0.04	-0.26	-2.83	0.006	-0.23–0.04
Occupation	0.26	0.48	0.04	0.55	0.583	-0.69-1.22
Comorbidity	1.29	0.98	0.11	1.31	0.191	-0.65-3.25
Family Monthly Income	-2.10	0.65	-0.30	-3.19	0.002	-3.41–0.79

Note: R² = 0.31

## Discussion

The findings reveal that RA patients experience a moderate level of SPB, with 40.38% reporting a significant burden. The most concerning aspect for patients was the worry about imposing excessive responsibilities on their caregivers, highlighting the emotional strain associated with the disease. Statistically significant predictors of SPB included marital status, Activities of Daily Living, and family monthly income. These findings underscore the impact of personal relationships, functional capacity, and economic stability on disease-related burden. The study’s importance lies in its patient-centered perspective, offering actionable insights for healthcare providers and policy development.

### SPB in RA patients

Chronic conditions, such as hypertension, diabetes, and stroke, are known for their intricate origins, enduring nature, and profound effects on life quality. The World Health Organization’s Global Burden of Disease study highlights that chronic diseases account for nearly 70% of global mortality [18, 19]. RA, characterized by symmetrical polyarthritis, poses a significant risk of joint deformities if intervention is delayed or inadequate, subsequently impairing daily functioning and life quality. In addition, the systemic impact of RA can extend to critical organ systems, potentially escalating to life-threatening complications. The results of this survey indicate that RA patients experience a moderate level of SPB, aligning with the observations from Zhang Meng et al.’s multi-center study on chronic disease patients [20]. RA patients’ primary concern was the fear that their caregivers assume excessive responsibilities. This was closely followed by feelings of being a substantial burden to caregivers and the belief that they impose financial strain on them. These concerns stem from the dependency on caregivers during active disease phases, due to the inability to perform routine tasks independently. The financial demands of long-term medication and regular medical evaluations for RA also contribute significantly to the perceived burden.

### Analysis of factors affecting SPB in RA patients

#### Impact of marital status on SPB

This survey findings highlight that RA patients who are divorced or widowed bear the heaviest SPB, surpassing that of their unmarried or married counterparts. The presence of a spouse is identified as a pivotal pillar of familial support, substantially amplifying the overall support system. Such robust support is instrumental in fostering a positive outlook towards managing the illness. As corroborated by research, there exists an inverse relationship between the intensity of SPB and the quality of family supports [21]. Within the RA patient demographic, those who are married often find solace in the continuous care and assistance provided by their partners, which is particularly beneficial given the reduced Activities of Daily Living caused by joint pain and deformities [22]. Conversely, divorced or widowed individuals, who must depend on parents or offspring for support, are more susceptible to feeling like a burden. This sentiment is often exacerbated by the inconsistent and potentially disruptive nature of care provided by these alternative caregivers.

#### Impact of activities of daily living on SPB

Our survey data clearly demonstrate an inverse relationship between the Activities of Daily Living of RA patients and the burden they perceive, a correlation that is consistent with both national and global research [1, 23]. At the inception of RA or during periods of inadequate disease management, patients are confronted with intense, progressing to joint deformities and functional limitations, which profoundly disrupt their daily activities [24]. This often necessitates a reliance on family members for support and care [22]. Prolonged dependency can engender a sense of guilt and self-reproach, thereby intensifying the perceived burden. On the flip side, patients who maintain effective disease control, enjoy pain-free joints, and preserve normal functional abilities encounter less interference in their day-to-day routines, consequently experiencing a reduced or even nullified sense of SPB [13].

#### Impact of economic status on SPB in RA patients

The variance analysis outcomes highlight the disparate effects of household monthly income on the SPB of RA patients. Notably, those earning less than 1,000 RMB per month are found to carry the heaviest burden, whereas individuals with a monthly income ranging from 3,000 to 4,000 RMB exhibit the lowest levels of perceived burden. The enduring and cyclical character of RA, compounded by the need for sustained medication and periodic medical evaluations, lays a substantial financial strain on the patient’s household [25, 26]. A shortfall in income can intensify this strain, thereby elevating the SPB. This correlation between economic constraints and perceived burden is in line with observations from studies on other chronic disease cohorts [1, 27]. In contrast, the study indicates that RA patients with a monthly income between 3,000 and 4,000 RMB tend to experience the lowest levels of SPB. This phenomenon can be largely attributed to the enhanced healthcare insurance system in China, which ensures widespread medical coverage, thereby reducing out-of-pocket expenses to a manageable level for this income group. However, a counterintuitive increase in SPB is observed among patients earning more than 4,000 RMB monthly, as opposed to those within the 3,000 to 4,000 RMB range. This anomaly could be attributed to various factors, including the patient’s age and societal standings [28, 29]. Patients who are in their prime earning years and serve as the primary financial support for their families might feel an amplified sense of burden due to the enduring financial demands of chronic disease management, including lifelong medication regimens.

This study, while providing valuable insights into the SPB among patients with RA, has several limitations that are worth noting. Firstly, the cross-sectional design of the study limits the ability to establish causal relationships between the SPB and the influencing factors identified. Secondly, the convenience sampling method used may not fully represent the broader RA patient population, potentially introducing selection bias. Thirdly, the study’s focus on patients from a single hospital, may restrict the generalizability of the findings to other settings or regions. Lastly, this study did not account for potential confounding factors such as the specific RA treatment regimens, disease severity, or psychological comorbidities, which could also significantly influence the SPB. Future research employing longitudinal designs, diverse and representative sampling, and comprehensive consideration of additional variables is needed to further validate and expand upon these findings.

## Conclusion

In conclusion, RA patients frequently encounter SPB, with almost half reporting moderate levels, significantly impacted by marital status, Activities of Daily Living, and household income. These insights underscore the urgency for multifaceted clinical interventions that not only enhance physical function via exercise but also attend to patients’ psychological wellbeing. Future endeavors should endeavor to prolong study durations, broaden sample scopes, and delve into additional contributory factors, thereby fostering a deeper comprehension of the self-perceived burden experienced by RA patients.

## Data Availability

All data generated or analysed during this study are included in this published article.

## Abbreviations

RA: Rheumatoid arthritis
SPB: Self-perceived burden
SPBS: SELF-perceived burden scale
ACR/EULAR: American college of rheumatology/European league against rheumatism
BI: Barthel index
